# Familial acute necrotizing encephalopathy temporally associated with SARS-CoV-2 infection: Two case reports and literature review

**DOI:** 10.1097/MD.0000000000050329

**Published:** 2026-08-21

**Authors:** Ning Zhang, Yipan Fan, Weixing Ge, Zhao Lin

**Affiliations:** a Department of Intensive Care Medicine, The Affiliated Jiangning Hospital of Nanjing Medical University, Nanjing, Jiangsu, China.

**Keywords:** acute necrotizing encephalopathy, case report, COVID-19, genetic susceptibility, MRI, *RANBP2*

## Abstract

**Rationale:**

Acute necrotizing encephalopathy (ANE) is a rare but life-threatening parainfectious encephalopathy characterized by rapid neurological deterioration following viral infections. Although most cases occur sporadically, familial clustering is uncommon and suggests the involvement of genetic susceptibility factors. The emergence of coronavirus disease 2019 (COVID-19) has broadened the spectrum of viral triggers associated with ANE; however, familial COVID-19–associated ANE remains rarely reported.

**Patient concerns:**

We retrospectively reviewed 2 siblings who developed ANE following laboratory-confirmed severe acute respiratory syndrome coronavirus 2 (SARS-CoV-2) infection in December 2022. The first patient was an 18-year-old woman who presented with seizures and progressive impairment of consciousness 2 days after COVID-19 infection. Her 24-year-old brother developed a similar neurological syndrome after SARS-CoV-2 infection, characterized by altered mental status and rapid clinical deterioration.

**Diagnose:**

Brain magnetic resonance imaging (MRI) of the female patient demonstrated typical bilateral symmetrical lesions involving the thalami, basal ganglia, corpus callosum, cerebellum, and cerebral white matter. MRI of her brother revealed bilateral symmetrical lesions predominantly affecting the lenticular and caudate nuclei. Genetic analysis showed no pathogenic variants in the RANBP2 gene. After comprehensive exclusion of alternative diagnoses, including other infectious encephalitis, metabolic disorders, autoimmune encephalitis, and toxic encephalopathy, both patients were diagnosed with COVID-19–associated ANE.

**Interventions:**

Both patients received immunomodulatory therapy, including high-dose corticosteroids and intravenous immunoglobulin. Supportive treatments and intensive neurological monitoring were also provided according to clinical conditions.

**Outcomes:**

Following treatment, both patients achieved partial neurological recovery; however, residual neurological impairment remained during short-term follow-up. The clinical course and neuroimaging findings were consistent with previously reported characteristics of ANE.

**Lessons:**

These familial cases highlight a potential association between SARS-CoV-2 infection and the development of ANE and suggest that genetic susceptibility factors other than RANBP2 mutations may contribute to disease pathogenesis. Early recognition of neurological deterioration, prompt brain MRI evaluation, and timely initiation of immunomodulatory therapy may improve clinical outcomes. Further genetic and mechanistic studies are warranted to identify additional susceptibility genes involved in COVID-19–associated ANE.

## 1. Introduction

Acute necrotizing encephalopathy (ANE) is a rare, rapidly progressive encephalopathy characterized by acute impairment of consciousness, seizures, and distinctive bilateral symmetrical brain lesions, predominantly involving the thalami, brainstem, cerebellum, and cerebral white matter.^[1]^ It is generally regarded as a parainfectious disorder triggered by viral infections rather than a consequence of direct viral invasion of the central nervous system.^[2]^ Although the influenza virus remains the most frequently reported trigger, increasing evidence indicates that SARS-CoV-2 infection may also be associated with the development of ANE.

Since the COVID-19 pandemic, an increasing number of COVID-19-associated ANE cases have been reported in both pediatric and adult patients. Most published reports describe sporadic cases, while familial occurrence remains exceptionally rare.^[3]^ Recent systematic reviews have suggested that COVID-19-associated ANE shares many clinical and radiological characteristics with classical ANE, although its pathogenesis remains incompletely understood.^[4]^ Excessive cytokine release, blood–brain barrier disruption, and immune-mediated injury are considered the principal mechanisms underlying neurological damage.

Genetic susceptibility is increasingly recognized as an important contributor to ANE. Pathogenic variants in *RANBP2* are the best-established genetic risk factors for familial or recurrent ANE.^[5]^ However, many patients with familial clustering do not harbor detectable *RANBP2* mutations, suggesting that additional susceptibility genes remain to be identified.

We describe 2 siblings who developed ANE shortly after laboratory-confirmed SARS-CoV-2 infection. Despite typical clinical manifestations and characteristic neuroimaging findings, both patients tested negative for pathogenic *RANBP2* variants. These cases expand the current spectrum of COVID-19-associated ANE and further support the possibility that genetic susceptibility beyond *RANBP2* may contribute to disease development. We also review the relevant literature to improve clinicians’ awareness of this rare but potentially fatal neurological complication.

## 2. Case presentation

Case 1: An 18-year-old Han Chinese woman, with no documented history of neurological disease, autoimmune disorders, chronic medical conditions, or prior episodes of encephalopathy, presented with fever and cough following laboratory-confirmed SARS-CoV-2 infection. Review of her available medical records revealed no family history of neurological disorders prior to this episode.

**Figure 1.**
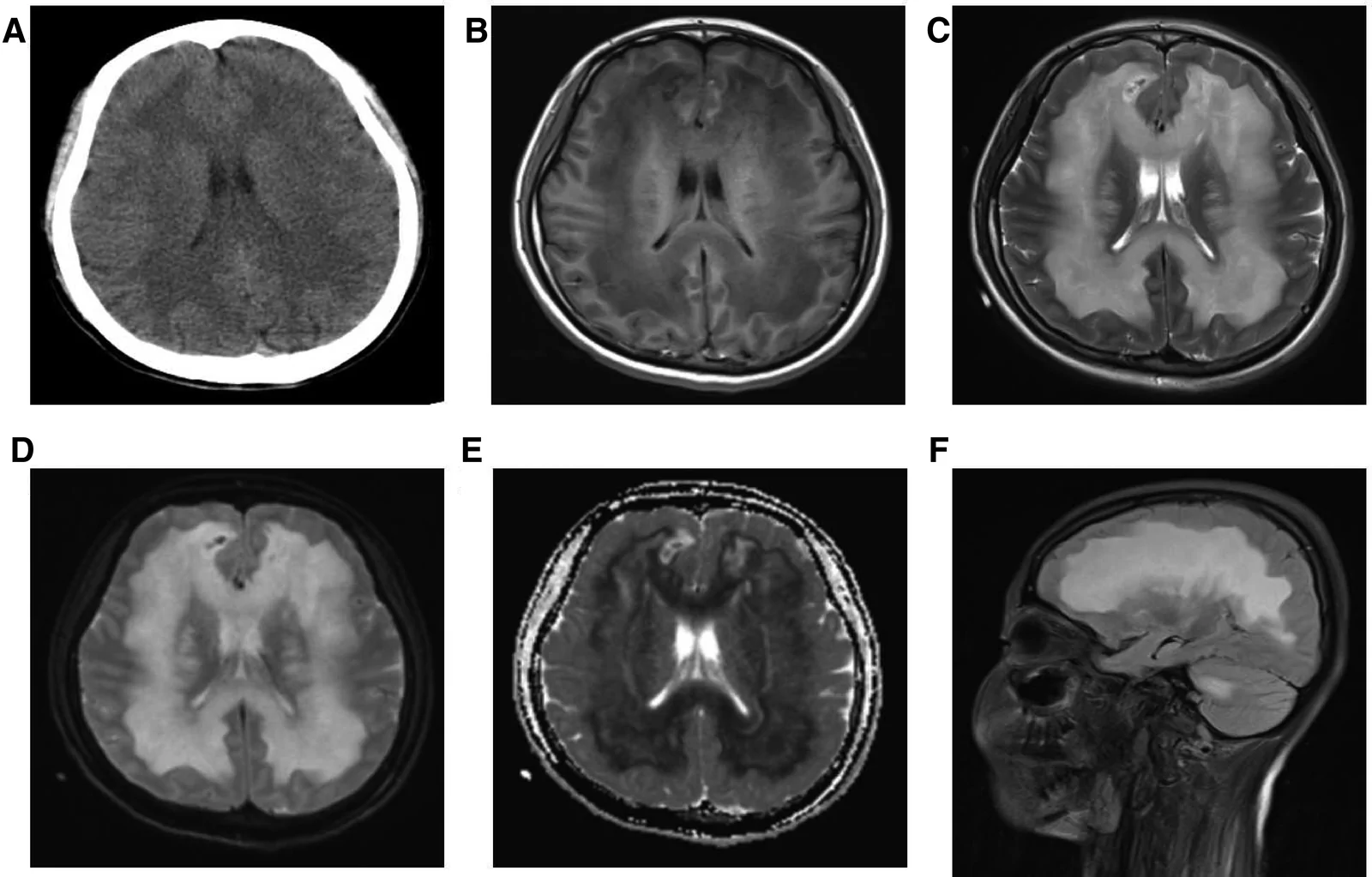
Neuroimaging findings in Case 1. (A) Initial non-contrast computed tomography (CT) showed no acute intracranial abnormalities. (B) Coronal T1-weighted image (T1WI), (C) coronal T2-weighted image (T2WI), (D) diffusion-weighted imaging (DWI), and (E) apparent diffusion coefficient (ADC) map demonstrated bilateral symmetrical lesions involving the thalami, lenticular nuclei, corpus callosum, and cerebral white matter. (F) Sagittal T2WI demonstrated additional involvement of the cerebellum and cerebral white matter.

Two days after the onset of respiratory symptoms, she developed generalized seizures followed by progressive impairment of consciousness. An initial non-contrast head computed tomography (CT) scan – performed at a local hospital – revealed no acute intracranial abnormalities (Fig. 1A). Given persistent neurological deterioration, she was transferred to our institution for comprehensive evaluation.

On admission, she was unconscious, exhibiting rightward gaze deviation, generalized hypertonia, and bilateral positive Babinski signs. Her Glasgow Coma Scale (GCS) score was E2V1M2 (total: 5/15), consistent with severe impairment of consciousness. Due to tachypnea and tachycardia, endotracheal intubation and mechanical ventilation were initiated following sedation.

Routine laboratory investigations showed elevated blood lactate levels, without evidence of marked systemic inflammation. Cerebrospinal fluid (CSF) analysis demonstrated a normal leukocyte count (1 × 10^6^/L), protein concentration (0.4 g/L), and glucose level (3.8 mmol/L). The Pandy test and microbiological studies – including bacterial, viral, and fungal assays – were all negative (Table 1).

**Table 1:** The results of cerebrospinal fluid analysis.

	Case 1	Case 2
Routine examination		
Color	colorless	colorless
Transparency	transparent	transparent
Pandy test	negative	negative
Leukocyte count (/L)	1 × 10^6	3 × 10^6
Biochemical test		
Total protein (g/L)	0.4	0.6
Glucose (mmol/L)	3.8	3.43
Chlorine (mmol/L)	123.2	124.3
Microbiological investigations		
Bacteria	negative	NA
Fungus	negative	NA
DNA virus	negative	NA
RNA virus	negative	NA

**Reference ranges:** All laboratory reference intervals are those routinely used at our institution.

CMV = cytomegalovirus, CSF = cerebrospinal fluid, DNA = deoxyribonucleic acid, EBV = Epstein–Barr virus, HSV = herpes simplex virus, PCR = polymerase chain reaction, RNA = ribonucleic acid.

Brain magnetic resonance imaging (MRI), performed on hospital day 5, revealed characteristic bilateral, symmetrical lesions involving the thalami, lenticular nuclei, corpus callosum, cerebellum, and cerebral white matter (Fig. 1B–F). Genetic testing for pathogenic variants in *RANBP2* was negative (Figure S1, Supplemental Digital Content 1). Based on the distinctive clinical presentation, characteristic neuroimaging findings, and exclusion of alternative etiologies, COVID-19-associated ANE was deemed the most probable diagnosis.

The patient received high-dose intravenous methylprednisolone (0.5 g/day for 3 consecutive days), intravenous immunoglobulin (20 g/day for 5 days), and supportive intensive care. Her level of consciousness gradually improved during hospitalization. At discharge, she remained neurologically impaired and was transferred for rehabilitation. Long-term follow-up data were unavailable.

Case 2: The patient’s 24-year-old brother developed fever and upper respiratory tract symptoms during the same timeframe and subsequently tested positive for SARS-CoV-2. Shortly thereafter, he exhibited progressive neurological deterioration, manifesting as impaired consciousness. Review of his medical records revealed no documented history of chronic neurological disorders or prior episodes of encephalopathy.

CSF analysis showed a normal leukocyte count (3 × 10^6^/L), protein concentration (0.6 g/L), and glucose level (3.43 mmol/L). The Pandy test and comprehensive microbiological investigations – including bacterial, viral, and fungal assays – were all negative (Table 1).

**Figure 2.**
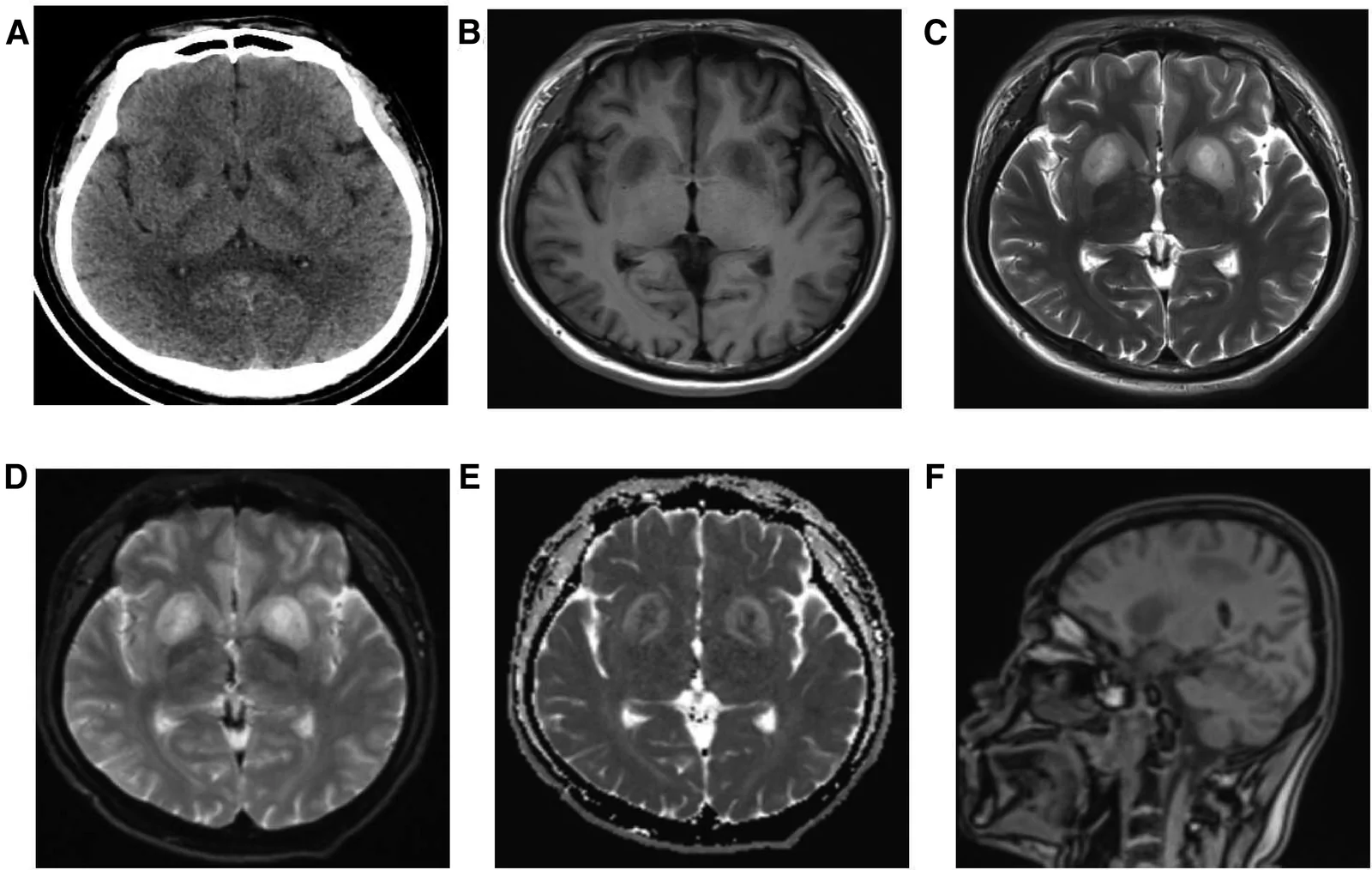
Neuroimaging findings in Case 2. (A) Brain computed tomography (CT) demonstrated bilateral lesions involving the lenticular and caudate nuclei. (B) Coronal T1-weighted image (T1WI), (C) coronal T2-weighted image (T2WI), (D) diffusion-weighted imaging (DWI), and (E) apparent diffusion coefficient (ADC) map confirmed bilateral symmetrical involvement of the lenticular and caudate nuclei. (F) Sagittal T1-weighted image demonstrated persistent lesions involving the basal ganglia.

CT demonstrated bilateral lesions involving the lenticular and caudate nuclei (Fig. 2A). Brain MRI revealed bilateral, symmetrical lesions involving the lenticular and caudate nuclei – radiological findings consistent with ANE (Fig. 2B–F). As in his sister’s case, genetic testing did not identify pathogenic variants in *RANBP2*, a gene associated with susceptibility to ANE.

The patient received multimodal therapy, including high-dose intravenous methylprednisolone, intravenous immunoglobulin, antiviral agents, and comprehensive supportive care. His level of consciousness gradually improved; however, at discharge, he remained nonambulatory and was referred for specialized neurological rehabilitation. Long-term functional outcomes were not available for follow-up.

Differential diagnosis: Several alternative diagnoses were considered during the diagnostic evaluation. Viral encephalitis was regarded as less likely because CSF analysis showed normal cell counts and protein concentrations, and microbiological investigations were negative. Autoimmune encephalitis was considered unlikely owing to the absence of characteristic clinical manifestations and supportive laboratory findings. Acute disseminated encephalomyelitis was excluded because the MRI findings demonstrated the typical bilateral symmetrical thalamic and basal ganglia lesions characteristic of ANE rather than asymmetric multifocal demyelinating lesions. Toxic-metabolic encephalopathy was also considered unlikely because metabolic investigations were unremarkable and the neuroimaging findings were highly characteristic. Collectively, the clinical presentation, neuroimaging features, and exclusion of competing diagnoses supported the diagnosis of COVID-19-associated ANE.

## 3. Discussion

ANE is a rare but life-threatening encephalopathy characterized by rapidly progressive neurological deterioration and distinctive bilateral symmetrical brain lesions.^[6,7]^ Although the influenza virus remains the most common infectious trigger, accumulating evidence suggests that SARS-CoV-2 infection may also be associated with the development of ANE. Recent systematic reviews indicate that COVID-19-associated ANE has been reported in both children and adults, although familial occurrence remains exceptionally uncommon.^[8,9]^ To our knowledge, reports describing familial COVID-19-associated ANE with negative *RANBP2* testing remain extremely rare.

The diagnosis of ANE in our patients was supported by the characteristic clinical presentation, typical MRI findings, normal CSF analysis, and exclusion of alternative neurological disorders. Differential diagnoses, including viral encephalitis, autoimmune encephalitis, acute disseminated encephalomyelitis, and toxic-metabolic encephalopathy, were carefully considered. However, the absence of CSF pleocytosis, negative microbiological investigations, characteristic bilateral symmetrical thalamic and basal ganglia lesions on MRI, and the clinical course strongly favored a diagnosis of ANE.

The pathogenesis of ANE remains incompletely understood. Current evidence supports an immune-mediated mechanism rather than direct viral invasion of the central nervous system.^[10]^ Excessive cytokine release, blood-brain barrier disruption, endothelial injury, and secondary neuroinflammation have all been proposed as major contributors to disease development. The absence of detectable pathogens in the CSF of our patients further supports this hypothesis.

Familial clustering represents one of the most intriguing aspects of the present report. Although pathogenic variants in *RANBP2* are the best-established genetic susceptibility factor for familial or recurrent ANE,^[11]^ both of our patients tested negative for known pathogenic *RANBP2* variants. This finding suggests that additional susceptibility genes or unidentified genetic mechanisms may contribute to disease development. Similar observations have been reported in a small number of familial ANE cases without detectable *RANBP2* mutations, indicating that the genetic architecture of ANE is likely more complex than previously recognized.^[12]^

Compared with previously published COVID-19-associated ANE cases, our report is distinguished by the occurrence of ANE in 2 siblings during the same SARS-CoV-2 outbreak. The similar clinical manifestations, neuroimaging findings, treatment responses, and absence of *RANBP2* mutations raise the possibility of shared genetic susceptibility or common environmental influences. Although causality cannot be established from two observational cases, these findings broaden the current clinical spectrum of COVID-19-associated ANE.

The neurological prognosis of ANE remains guarded. Previous studies have demonstrated that survivors frequently experience persistent cognitive impairment, motor dysfunction, epilepsy, or other neurological sequelae despite aggressive immunomodulatory therapy.^[13]^ Early recognition, prompt neuroimaging, and timely initiation of high-dose corticosteroids and intravenous immunoglobulin remain the cornerstones of current management and may improve neurological outcomes.

Several limitations should be acknowledged. First, this study included only 2 patients from a single family, limiting the generalizability of our findings. Second, comprehensive genomic analyses, including whole-exome or whole-genome sequencing, were not available; therefore, additional susceptibility variants could not be investigated. Third, long-term neurological follow-up was limited, preventing a comprehensive evaluation of functional recovery. Future multicenter studies integrating detailed genomic analyses and long-term follow-up are warranted to further clarify the relationship between SARS-CoV-2 infection, genetic susceptibility, and ANE.

In conclusion, we report 2 rare familial cases of COVID-19-associated ANE with negative *RANBP2* testing. These cases suggest a possible association between SARS-CoV-2 infection and ANE in genetically susceptible individuals rather than establishing a direct causal relationship. Clinicians should maintain a high index of suspicion for ANE in patients presenting with rapidly progressive neurological symptoms following SARS-CoV-2 infection. Early neuroimaging evaluation and timely immunomodulatory therapy may improve clinical outcomes. Further genomic studies and long-term follow-up are needed to better understand the genetic basis and clinical course of familial COVID-19-associated ANE.

## Author contributions

**Writing – original draft:** Ning Zhang, Yipan Fan.

**Investigation:** Weixing Ge.

**Writing – review & editing:** Zhao Lin.


